# Systemic Front Line Therapy of Follicular Lymphoma: When, to Whom and How

**DOI:** 10.4084/MJHID.2016.062

**Published:** 2016-11-07

**Authors:** Francesca Pavanello, Sara Steffanoni, Michele Ghielmini, Emanuele Zucca

**Affiliations:** Istituto Oncologico della Svizzera Italiana, Ospedale San Giovanni 6500 Bellinzona, Switzerland

## Abstract

The natural history of follicular lymphoma is usually characterized by an indolent course with a high response rate to the first line therapy followed by recurrent relapses, with a time to next treatment becoming shorter after each subsequent treatment line. More than 80% of patients have advanced stage disease at diagnosis. The time of initiation and the nature of the treatment is mainly conditioned by symptoms, tumor burden, lymphoma grading, co-morbidities and patients preference. A number of clinical and biological factors have been determined to be prognostic in this disease, but the majority of them could not show to be predictive of response to treatment, and therefore can’t be used to guide the treatment choice. CD20 expression is the only predictive factor recognized in the treatment of FL and justifies the use of “naked” or “conjugated” anti-CD20 monoclonal antibodies as a single agent or in combination with chemo- or targeted therapy. Nevertheless, as this marker is almost universally found in FL, it has little role in the choice of treatment. The outcome of patients with FL improved significantly in the last years, mainly due to the widespread use of rituximab, autologous and allogeneic transplantation in young and fit relapsed patients, the introduction of new drugs and the improvement in diagnostic accuracy and management of side effects. Agents as new monoclonal antibodies, immuno-modulating drugs, and target therapy have recently been developed and approved for the relapsed setting, while studies to evaluate their role in first line treatment are still ongoing. Here we report our considerations on first line treatment approach and on the potential factors which could help in the choice of therapy.

## Introduction

Follicular lymphoma (FL) represents 70% of all indolent non Hodgkin lymphomas with an increasing incidence in western countries over last two decades. More frequently FL is diagnosed in asymptomatic advanced stage patients. The clinical course is heterogeneous, characterized by a high response rate to first line therapy and by subsequent relapses that need subsequent therapy lines or eventually, a re-challenge with previous lines. The advent of biological agents as rituximab (R), the improvement of supportive care and availability of different chemotherapy (CT) regimens improved the prognosis of FL in the last decades,^1^–^5^ with an expected median survival nowadays approaching or even exceeding 20 years, particularly in the younger patients.^6^,^7^ Early disease progression and transformation into high grade lymphoma remain the major reasons for shorter survival.^8^ Classically, and according to most guidelines, the decision of when to treat and which treatment regimen to use is made considering stage (early versus advanced), tumor burden (low or high) and patients symptoms: in this review we also address others factors that could as well influence the decision making. Since patients with FL usually have a long life expectancy and the course of FL is characterized by recurrent relapses, the choice of the treatment regimen and its starting time should be made considering not only overall response rate (ORR) and time to progression, but also its impact on quality of life (QoL), future therapies (including transplantation), risk of transformation into aggressive lymphoma and risk of long-term side effects, like therapy-related secondary malignancies.^9^

Variables which could potentially influence the choice of treatment in FL include:

- Clinical prognostic scores: baseline FLIPI (Follicular Lymphoma International Prognostic Index) and FLIPI2 are validated as prognostic risk factors for early and advanced FL in clinical trial settings and outside of clinical trials, irrespectively to CT, R-based and immuno-chemotherapy-based treatment.^10^ More recently, the m7-FLIPI score, combining clinical parameters (FLIPI and Performance status according to ECOG score) with gene mutations (EZH2, ARID1A, MEF2B, EP300, FOXO1, CREBBP, and CARD11), demonstrated a prognostic value in FL patients receiving first-line immuno-chemotherapy (ICT).^11^ Nevertheless, at present, no study has yet shown that patients of different clinical risk categories need different treatments. Since patients with higher FLIPI score have a higher risk of relapse,^12^ it could be tempting to intensify the systemic treatment in these patients, but so far this has only shown to improve progression free survival (PFS) and not overall survival (OS).^13^
- Tumor burden: for patients with advanced stage, the British and the French study groups defined clinical criteria to identify patients with high tumor burden (HTB) (Table 1), for whom an immediate treatment should be recommended. For patients with HTB, the treatment goal is primarily the response rate, in order to obtain as soon as possible a relief of symptoms and a longer PFS. In this situation, an immediate local treatment as palliative radiotherapy (RT) and/or systemic therapy is highly recommended. Asymptomatic patients who do not fulfill all criteria can be defined as having low tumor burden (LTB) and do not generally require any immediate treatment.
- Bulky disease: despite several trials recognizing tumor bulk as a poor prognostic factor for response and survival,^14^ there has not yet been a worldwide consensus on how to define the “bulky disease”^13^,^15^,^16^ and how to treat FL patients with a large tumor mass. The frequent practice of consolidation with involved-field radiotherapy (IFRT) after ICT is not supported by randomized studies. Only a retrospective analysis, performed in advanced stage bulky FL, failed to demonstrate significant differences in terms of ORR, PFS and OS between patients treated with consolidation IFRT and those did not.^15^ Given that the 2014 European Society for Medical Oncology (ESMO) guidelines advice the use of systemic treatment in early stage patients with adverse prognostic factors, patients with bulky disease may be treated with systemic approach even in early stages.^17^
- Age and sex: pediatric-type FL is a new separate disease in the 2016 revision of the World Health Organization (WHO) classification,^18^ and will not be considered in this discussion. In non pediatric patients, three large analysis were performed demonstrating that increasing age has a negative prognostic value: in one study patients younger than 40 years had a favorable prognosis compared to older ones;^19^ in the other two, follicular lymphoma-specific survival was similar for cases < 40 years and in those aged 40–60.^6^,^7^ All studies concluded that younger patients have a similar or better prognosis than older patients, and there is, therefore, no need to adopt for them a more aggressive approach. In some prospective trials, gender was recognized as a prognostic factor in FL patients treated with R given either upfront^20^ or as maintenance (RM).^21^ Females, particularly of older age, seem to have a better response quality (regarding remission quality and PFS) and this appears to be related to a lower clearance of the drug compared to men.^20^ Based on the available data it is not yet justified to base treatment decisions on gender or age, although fertility preservation issues should be discussed with the patients in the decision making.
- Grading: most studies on the prognostic and predictive role of histological grade were conducted in the pre-Rituximab era, they enrolled a small number of patients and had a relatively short follow-up. Moreover, the lack of reproducibility among pathologists on the evaluation of grade makes the interpretation of international data even more difficult.^22^,^23^ The 2008 WHO classification recognises three grades of FL according to the number and distribution of centroblasts (0–5, 6–15, and > 15 per high-power field, respectively). Grade 3 is further subdivided into 3A (centrocytes still present) and 3B (sheets of centroblasts). Differences in molecular, genetic and clinical behavior suggest that FL G1 and G2 have an indolent course while G3 has a more aggressive course. While FL G1 and G2 is applied a classical FL treatment algorithm (according to stage and tumor burden), FL G3B is usually treated following a Diffuse Large B-Cell Lymphoma (DLBCL) algorithm, due to many histological, biological and clinical similarities among the two entities. Some studies with a follow-up longer than five years demonstrated a potential curability of patients with FL G3B receiving an anthracycline–containing regimen.^24^,^25^ For FL G3A there is more controversy, as some would treat them as FL, and others as DLBCL. Most studies, reporting the outcome of patients with FL G3A, were retrospective and not randomized, and their results and conclusions are not unanimous. The distinction between FL G3A and FL G3B is not always possible because the studies were performed before the publication of the 2008 WHO classification. Most studies failed to show a survival benefit^22^,^24^ and potential curability in long-term FL G3A survivors after anthracycline-based CT^23^,^24^,^26^ (Table 2) as reported for FL G3B. A retrospective Swedish study on a cohort of 505 patients with long follow-up showed that patients with advanced stage FL G3A have a similar outcome to FL G1–2 regardless if anthracycline were administered or not,^24^ hence, they may be treated similarly.^27^ No data are available on RT efficacy in early stage FL G3A, thus a systemic treatment is advised, particularly in bulky or high FLIPI score patients.^17^
- Genetic features, microenvironment, and serum factors: the t(14;18) with BCL2 rearrangement is present in up to 90% of the nodal FL G1–2 but is less frequent in FL G3B. High levels of circulating t(14;18) can be found in healthy individuals and can predict the onset of FL some years earlier.^28^ A recent study, performed by German Low-grade Lymphoma Study Group, showed that FL with and without BCL2 breaks had no differences in survival outcome.^29^ The last WHO classification also identifies a distinctive subtype of t(14;18)-negative nodal FL (diffuse-appearing FL) characterized by deletion 1p36. It is usually confined to the inguinal region with an indolent course and its standard treatment strategy is unknown.^30^ Positivity of BCL6 rearrangements and Myc abnormalities are associated with negative outcome. The acquisition of Myc positivity in FL might identify a biological transformation into a more aggressive disease deserving a specific treatment approach. Others genetic factors (mutTP53, mutMLL2, mutEZH2 and delCDKN2A), microenvironment gene expression profile (genes related to T-cell and macrophage activation)^31^ and serum chemokines (IL-2R, IL-1RA, and CXCL9)^32^ are recognized to be associated with a poor prognosis in FL. However, all these factors need to be confirmed in prospective clinical trials before being applied for risk stratification, and risk adapted treatment in daily clinical practice.
- Primary extra-nodal localization: duodenal-type FL and primary cutaneous follicle centre lymphoma are molecularly distinct entities with specific characteristics, excellent prognosis and indolent course but, due to their rarity, there is not a worldwide consensus on their optimal treatment. Cutaneous follicle centre lymphomas can be treated with local RT.^33^ Duodenal-type FL displays excellent prognosis with watchful waiting (WW) approach if asymptomatic or after complete resection, therefore, it seems justified to delay a systemic treatment until symptomatic disease progression.^34^ Recently, a study demonstrated that also RT could be effective and safe in this setting.^35^ In both cutaneous and duodenal localizations, systemic front-line treatment (R alone or ICT) is only required in symptomatic patients with extensive and multifocal disease.
- Risk of transformation: Histologic transformation (HT) to an aggressive lymphoma is a well-described event in the natural history of FL. However, it remains unclear if there is already a predisposition to HT and whether this can be detected at the time of diagnosis.^36^ The risk of HT into aggressive lymphoma is approximately of 3% per year during the first ten years and seems correlated with advanced stage and high IPI and FLIPI scores.^37^ These scores merely predict a poorer survival, reflect tumor load and clinical characteristics but are not markers of the individual susceptibility to transformation.^36^ A more important point is whether early systemic treatment could influence the HT risk. This remains an open question as data on this issue are limited and controversial. Patients who were initially observed had an unexpectedly higher rate of transformation compared to those treated upfront with systemic therapy in the St Bartholomew’s series^37^ but not in other retrospective studies. In our series, patients receiving CT have a higher risk of HT compared to patients in WW or treated with R alone;^38^ similar results were also reported by an epidemiologic study from the Mayo Clinic.^39^ The randomized studies comparing expectant management at diagnosis with immediate treatment do not help clarify this issue since data on HT are reported only by two studies conducted more than two decades ago: a GELA trial found no differences in the risk of HT,^40^ while in a study of the National Cancer Institute, the patients randomly assigned to WW appeared to carry a higher risk of HT.^41^ This latter study, however, has never been fully published. The possible influence of the type of initial chemotherapy on the likelihood of transformation is also controversial: a retrospective trial concluded that initial treatment with anthracyclines may play a preventive role,^42^ but in another retrospective study the HT rate did not change according to whether patients had received doxorubicin or not.^43^ The role of RM in preventing transformation remains also unsolved: in the PRIMA study there was no significant difference in the risk of transformation between patients who received RM versus those who did not. 44 These data was later confirmed by Ardeshna.^45^ On the other hand, in the National LymphoCare study patients on RM had a lower incidence of transformation.^46^ In conclusion, now there is no clear evidence to suggest any strategy in order lower the risk of subsequent HT.
- Positron Emission Tomography (PET): FL of any grade is fluorodeoxyglucose (FDG) avid,^47^–^49^ and its FDG avidity is heterogeneous within and across patients. The SUVmax is widely variable (range 3 to 40) and does not seem to correlate with the risk of progression.^50^ However, there is increasing evidence of a correlation between the uptake intensity and the histological grade and ─ despite a certain degree of inconsistency in defining a SUVmax threshold─ FDG-PET/CT imaging can be an effective tool to detect histological transformation.^51^–^53^ Indeed, large cell transformation is most often focal, and it is associated with higher FDG uptake. Hence, to increase the chances of detecting an underlying transformation, diagnostic biopsies should, whenever possible, be directed to the site of greatest FDG avidity.^54^ It has been suggested that the emergence of a focal lymphoma site with SUVmax 3 times higher or more than the others on a single scan, or that has tripled its uptake on serial scans, raises suspicion of histological transformation and should be biopsied.^55^ As data have become available, analogous to diffuse large B cell lymphoma and Hodgkin lymphoma, response assessment with PET/CT has been found to be an independent prognostic factor for FL progression and overall survival.^56^–^58^ In a French prospective multicentre study of patients with HTB FL G1–3A treated with R-CHOP, without rituximab maintenance, the 2-years PFS was 51% and 87%, respectively, in patients PET-positive and PET-negative at the end of therapy.^58^ Similar results were retrospectively found in other two cooperative studies, conducted on patients prospectively enrolled in the PRIMA^56^ and the FOLL05^57^ trials. A pooled analysis of central scan reviews of these three large studies confirmed that PET/CT performed at the end of ICT induction (with R-CHOP, R-CVP, or R-FM), is highly predictive of both PFS and OS, particularly when the PET-response status is defined using a positivity cut-off of 4 (uptake moderately increased above the liver at any site) on the now recommended Deauville 5-Point Scale (5PS).^59^ Conventional CT-based response in this study was only weakly predictive of PFS and based on this evidence, the recent consensus guidelines of the ICML Imaging Working Group^60^ and the Lugano Classification^16^ recommended that PET-CT rather than contrast-enhanced CT scanning should be considered as a new standard for initial staging and response assessment of FL. In the previously mentioned French cooperative study the response assessed by PET/CT after four courses of R-CHOP was also highly prognostic.^58^ However, the role of PET/CT during treatment (interim-PET) is less well established and is not currently recommended. These data can be extremely important for the clinical practice since end-of-therapy PET can identify FL patients whose disease has no longer an indolent course with increased risk of progression or death.^59^ Moreover, there is preliminary evidence suggesting that functional PET parameters such as the baseline metabolic tumor volume (MTV) can be used as biomarkers for the development of first-line PET-adapted approaches in FL.^61^ Unfortunately, the implication of PET results in the definition of treatment remains still poorly known and clinical studies to evaluate response-adapted strategies based on the results of PET/CT ─ possibly associated with assessment of minimal residual disease (MRD) ─ are highly warranted and represent a major challenge for clinical research in FL.^62^
- Assessment of MRD (detection of BCL2/IGH rearrangement): it is already demonstrated that patients with MRD persistence after transplantation have a worse outcome.^63^–^66^ However, the role of MRD assessment after standard ICT in FL remains very controversial. The available results of different studies are conflicting and have some limitations due to retrospective nature, small sample size, mixed tissue sources (peripheral blood versus bone marrow) and lack of prospective planning for MRD time points.^67^–^72^ Two studies of the Fondazione Italiana Linfomi (FIL) showed that MRD is an independent outcome predictor in patients with FL receiving rituximab-intensive programs.^73^,^74^ It has been suggested that monitoring MRD may be useful for starting preemptive therapies before a frank clinical relapse. On the other hand, maintenance may be unnecessary in patients with MRD negativity after induction treatment. To test this hypothesis, the FIL has launched the FOLL-12 phase 3 trial (NCT003170-60), which will evaluate in FL patients undergoing first-line therapy whether an FDG-PET and MRD response-based maintenance therapy can replace the standard RM therapy currently given to nearly all the patients responding to standard ICT.

All the above-mentioned prognostic factors are able to identify patients at high risk for early progression or death, but are not able to identify which patients need one or the other treatment strategy: currently the main parameters conditioning first line systemic treatment approach are still stage (limited versus advanced), tumor burden, grading of FL and presence of symptoms.

## Treatment

### Systemic front-line therapy for limited stage (stage I and II)

Stage I and II account for about 15–20% of FL and the median survival ranges up to 25 years from diagnosis. FL is highly radio-sensitive: RT achieves ORR of 90% if performed within one year from diagnosis and with a standard dose of 24 Gy in 12 fractions, without a higher risk of second malignancies.^75^ When the involved sites are contiguous and can be safely encompassed within a radiation field, local RT can induce long-term remissions in about 50% of the patients and is therefore considered curative in the limited stage. The other 50% of the patients relapse within ten years outside of the radiation field.^76^ Despite National Comprehensive Cancer Network (NCCN) and ESMO guidelines recommend IFRT as preferable first line strategy for non-bulky stage I and II FL, less than 40% of patients with limited stage are treated with RT alone in clinical practice.^77^ Observation only after an excisional biopsy is also a feasible strategy: a retrospective analysis found a 10-years OS of 85% with this approach and, after a median follow-up of 7 years, more than 60% of the patients did not require any therapy.^78^ Surgery alone could be considered in asymptomatic patients, who are not suitable for RT or systemic treatment due to short life expectancy or co-morbidities or in young women needing fertility preservation.

1. *Systemic treatment VERSUS radiotherapy.* No randomized trials comparing RT versus front-line systemic therapy are reported. The Lymphocare Study and other observational studies compared different approaches in early stage FLs: even if improved PFS was observed in patients treated with systemic treatment compared to those undergoing RT alone, no difference in OS was observed[79–82]. Since in limited disease both approaches (systemic and local treatment) seem not to have any survival prognostic value, the best approach should be the one with the best toxicity profile. Also favoring upfront RT, one study demonstrated a lower 10-years risk of transformation after RT in early stage disease (around 18%)^83^ compared to the general risk of transformation (ranges from 15 to 31%).^37^
2. *Systemic treatment WITH radiotherapy:* Since more than 50% of the patients treated with RT alone relapse outside the radiation field, the addition of systemic treatment to RT could reduce the risk of distant relapse. This combination does not seem to improve OS in older randomized trials and, moreover, it could result in overtreatment and be responsible for early and late side effects, unacceptable in this setting.^84^,^85^ The only prospective trial (from the MD Anderson Cancer Center) exploring efficacy and tolerability of Radio-chemotherapy (RCT) concluded that this strategy led to a better Disease Free Survival at 10 years compared to RT alone, but without advantage in OS, probably because of a higher incidence of secondary tumors and myelodisplastic syndromes.^86^ R may represent a less toxic systemic treatment alternative to CT and in vitro models provided evidence of significant synergism when it is associated with RT.^87^ Furthermore, R may reduce MRD with the improvement of duration response and survival.^68^ However, the available retrospective studies evaluating the outcome of R-RT combination showed improved disease control in terms of PFS for the combined strategy compared to RT alone, but no survival benefit was observed.^88^–^90^

### Systemic front-line therapy for advanced stage (stage III and IV)

About 80% of patients with FL are diagnosed with advanced stage. Despite the improved outcome, most patients still experience disease relapse at a median time of 1.5–5 years, depending on when the active therapy start^91^ and on the intensity of the treatment regimen used.^21^,^92^ A standard first line therapy for advanced stage FL has not yet been defined. The main decisions to take at presentation are when to start therapy and which regimen to choose. This strategy depends on the histological grade (see above) and the tumor burden according to BNLI or GELF criteria.

1. *Low tumor burden:* As discussed above, since FL is considered an incurable disease and in most cases has an indolent and chronic course, patients with LTB and no symptoms can be followed until they fulfil the criteria for initiation of treatment. In the pre-rituximab era retrospective and prospective studies had excluded a potential survival disadvantage when CT is differed until symptoms or organ failure risk in patients with LTB.^93^,^94^ WW delayed the exposure to CT and its potential side effects, with a better preservation of QoL. Spontaneous regressions (approximately 12% of patients) were observed in patients assigned to WW^45^ and approximately 40% of patients aged over 70 years, never needed any treatment and died of non-lymphoma related causes.^91^ More recently, a chemotherapy-free approach with single agent R, given with the weekly × 4 schedule, achieved an ORR of more than 70% in untreated LTB FL, with a range of complete response (CR) of 26–36%[95, 96] and a median PFS of 23.5 months becoming longer if R is continued.^97^,^98^ It was, therefore, tempting to evaluate if R alone could not substitute for WW in asymptomatic and LTB FL. A British randomized prospective study compared the outcome and QoL in the patients assigned to WW versus those to R. The patients treated with R upfront had a lower need of new treatment (46% versus 88%) and a better PFS (82% versus 36%) at 3 years, but this did not translate into a survival benefit (97% versus 94%). The QoL evaluation in this study showed that patients under the WW strategy were more worried about needing treatment and felt less control of their situation compared to patients treated with R upfront. Nevertheless, an initial surveillance strategy remains an acceptable approach for LTB FL patients, who do not want active therapy. How to perform WW approach remains object of discussion, clinical surveillance should be done every 3–4 months for the potential risk of rapid progression; radiological controls are recommended in case of new signs/symptoms that suggest progression disease.
2. *High tumor burden:* For patients with symptomatic advanced stage FL, various therapeutic options are available, ranging from single agent to multi-agents CT. The addiction of R to several CT regimens conferred an improvement in OS with a pooled HR for mortality of 0.63.^99^–^102^ The optimal regimen of CT, given in combination with R, in advanced stage FL patients remains controversial; however age, co-morbidity, and patient preference should play an important role in the decision making. None of the ICT regimens demonstrated superior survival in FL G1–3A patients; nevertheless R-CHOP and purine analogues containing regimens (R-FM) obtained a higher ORR (91–93% versus 88%) and a longer time to treatment failure (62–59% versus 46% at 3 years) and higher 3 years-PFS (68-63% versus 52%) compared to R-CVP, but caused a higher toxicity.^103^ An alternative ICT regimen is R plus bendamustine: its efficacy and toxicity in untreated advanced stage FL G1–2 were compared with those of R-CHOP or R-CVP in two randomised clinical trials.^92^,^104^ R-bendamustine obtained a higher CR (40 versus 30%), longer median PFS (69.5 versus 31.2 months) and time to next treatment (TTNT) (not reached versus 42.3 months) compared to R-CVP or R-CHOP, with fewer side effects.^92^ R as a single agent has as well been studied in patients in need of treatment. Given in the standard 4 weekly infusions as induction, it obtained an ORR of 81% with 31% of CR and a median Event Free Survival (EFS) of 19 months in CT-naive FL G1–3A patients; data that can be extrapolated from the SAKK 35/98 trial, where responders to induction were randomised to RM versus observation. Tumor burden was not formally assessed according to GELF criteria when patients were enrolled, however almost all cases had a stage higher than I (96%) and half of them had bulky disease at the moment of starting the ICT (53%). A higher response rate and a longer EFS were obtained with prolonged treatment, with an improvement of ORR to 92% and of median EFS to 36 months.^105^ If single agent R induction is to be used, the RESORT trial showed that RM fails to improve OS compared to observation or rituximab retreatment (RR): after a median follow-up of 3 years, the RR has not conferred any disadvantages regarding QoL and time to treatment failure. Nevertheless, RM strategy seems to confer a longer time to first CT, at the cost of an average use of R 3.5 times higher than with the RR strategy.^106^ Recently, numerous trials have explored the safety and efficacy of the combination of R plus other biological agents. One of the most promising agents is lenalidomide that showed a synergism of action in combination with R (R2) and a good profile of tolerability in the relapsed FL setting.^107^ The efficacy and tolerance of this combination were successively studied in untreated FL G1–3A patients.^108^,^109^ Approximately half of the enrolled patients (45.4%) had an advanced stage FL and approximately half of them meet GELF criteria for HTB(54%). The R2 regimen achieved in the subgroup of FL patients an ORR range of 93–98% (CR/uCR range of 72–87%) with PFS of 89% and 78.5% at 2 and 3 years, respectively.^108^,^109^ R2 in untreated FL G1–3a patients, strictly in need of treatment according to GELF criteria, was shown to achieved a higher CR/CRu rate than R alone (61% versus 36% in the independent radiological response review) in a randomised study at the expected cost of higher toxicity.^110^,^111^ Moreover, a significant advantage of the combination arm was shown regarding TTNT.^112^ Comparing the outcome after R2 regimen with those after ICT (as R-CVP, R-CHOP and R-FM),^103^ where three years-PFS ranges from 52 to 83%, the R2 regimen could be considered a possible alternative first-line treatment in FL patients, because probably better tolerated. An on-going randomised study is now comparing the outcome of R2 versus ICT in HTB FL patients (RELEVANCE trail).

The phase III GALLIUM study compared the efficacy and safety of obinutuzumab (new ant-CD20) plus chemotherapy (CHOP, CVP or bendamustine) followed by obinutuzumab maintenance with those of rituximab plus chemotherapy followed by R maintenance in untreated indolent NHL patients (85% of them with FL). Results from a pre-planned interim analysis showed that obinutuzumab-based treatment significantly improved PFS compared to R-based treatment.^113^

### Rituximab maintenance

A strategy of RM after first-line or after salvage therapy in relapsed patients was performed with the aim of improving PFS. A number of studies showed an improvement in disease control with RM after induction therapy with R alone,^98^,^105^,^114^ with CT^115^ and with ICT^21^ with an HR of 0.54 in the meta-analysis.^116^ The price to pay for this effect is a higher rate of infection-related adverse events (HR of 1.67). All these studies failed to prove a statistically significant OS benefit with RM after first-line induction therapy,^116^,^117^ although a favourable trend in OS was observed among patients receiving RM after salvage treatment.^117^ The schedules used for RM differ in various studies without any statistically significant effect on the outcome.^116^ In the studies that also enrolled patients in response or stable disease after first-line, RM was performed either with administrations every two months^21^,^105^,^114^ or with four weekly doses every six months.^114^,^117^ The different maintenance schedule is due to pharmacokinetic data, with detectable serum R ranging from 3 to 6 months after four infusions.^118^,^119^ The optimal duration of maintenance with R in patients who maintain a status of remission or response is also not definitely established: an improvement of median EFS of approximately 20 months (16 versus 36 months) was obtained with 1 year of RM compared to induction only (trial SAKK 35/98) going up to 8 years-EFS of 45% in responding, previously untreated patients.^97^,^105^ The SAKK 35/03 compared EFS after a short term RM (1 year) and after long term schedule with R every two months until a maximum of five years. After a median follow-up of 6.4 years, the difference in terms of EFS was ample (median EFS 3.4 versus 5.3 years), but insufficient to reach statistical significance and median OS was approximately of 8 years in both arms. Two years of RM is the currently preferred choice in the absence of a clear survival benefit with a longer term maintenance and because better tolerated (0% versus 11.6% of unacceptable toxicity).^98^

### Consolidation treatment

Since FL tends to relapse, consolidation with different approaches was studied for the advanced stage after response to induction therapy.

- Autologous stem cell transplantation. Three randomized trials before the R era^120^–^122^ and one after the advent of R^123^ compared the outcome of High Dose Therapy followed by Autologous Stem Cell Transplantation (ASCT) versus conventional CT in first line: in all studies ASCT achieved a longer PFS, without any benefit in OS. This result was confirmed by a Cochrane meta-analysis,^124^ which showed a trend toward increased risk of secondary malignancies in patients receiving ASCT. As a consequence, this approach is not justified as consolidation treatment after fist-line therapy outside of clinical trials.
- Radioimmunotherapy (RIT). RIT was used as front-line therapy in FL^125^–^128^ with interesting results, but its use as consolidation therapy is of potentially greater importance in clinical practice. Phase II trials demonstrated the feasibility, tolerability, and efficacy of this strategy after first line therapy with CT alone^129^,^130^ or R-containing therapy.^131^–^133^ In a randomized trial RIT with yttrium-90 (90Y) - ibritumomab tiuxetan (Zevalin) as consolidation after first line chemotherapy produced a longer PFS compared to observation alone, but without any survival benefit.^134^ This benefit was not confirmed by a subsequent randomized study.^135^ Nevertheless, both US Food and Drug Administration and European Medicines Agency approved 90Y-Ibritumomab tiuxetan as consolidation therapy in untreated FL patients in partial or complete response after first-line induction therapy. A randomized phase II trial, comparing RIT consolidation with RM, showed a higher PFS with RM than with RIT consolidation, without any difference in OS.^136^ Moreover, both strategies (RM and RIT consolidation) showed comparable incremental quality-adjusted life-years before the first progression, but a higher incidence of haematogical toxicity was observed in the RIT arm.^137^ Therefore consolidation with RIT after standard R-based induction therapy is not routinely performed.

## Figures and Tables

**Table 1:** Criteria for Low Tumor Burden definition

**BNLI criteria**

No rapid, generalized disease progression in the preceding three months
No life treating organ involvement
No evidence of renal or macroscopic liver infiltration
No bone lesions
No B symptoms or pruritus
Normal hematological function (Hemoglobin> 10g/dl, Platelets> 100×10^9/L, WBC count> 3×10^9/L)

**GELF criteria**

Normal serum concentration of Lactate dehydrogenase and beta2-microglobulin
Largest nodal or extra-nodal tumor lesion<7 cm
No more than 3 nodes in 3 distinct nodal areas with a diameter> 3 cm
No significant serous effusions
No risk of organ compression or compromise
No symptomatic spleen enlargement
No B Symptoms
Hemoglobin> 10g/dl, Platelets> 100×10^9/L, ANC>1.5×10^9/L

BNLI: British National Lymphoma Investigation; WBC: White Blood Cell; GELF: Group d’Etude des Lymphomes Folliculares. ANC: Absolute Neutrophil Count.

**Table 2:** Outcome of patients with Follicular Lymphoma after first line anthracyclines-based chemotherapy according to histological grade

Study	Population studied	Treatments	Median follow-up	Conclusions of the authors
Miller et al., 1997[26]	FLCL (N=389)	Anthracycline-based regimen (100%)	17 years	No plateau of the survival curve was achieved after anthracyclines-based regimens
Rodriguez et al, 2000[25]	FLCL (N=62)	Anthracycline-based regimen ± RT (76%) Non-anthracycline-based ± RT (13%) RT only (11%)	15 years	One third of the patients never relapsed, suggesting a potential curability for FLCL after anthracyclines–based treatments
Chau et al., 2003[22]	FL G1 (N=92 ) FL G2 (N= 68) FL G3A (N=44) FL G3B (N=11)	Anthracycline-based chemotherapy (27%) Non-anthracycline-based (38%) RT (22%) Surveillance (13%)	55mo for FLG1 57mo for FLG2 80mo for FLG3	No difference in terms of OS and FFS among FL grades 1–3 or between G3A and G3B First-line anthracyclines did not influence OS or FFS in patients with G3.
Hsi et al., 2004[138]	FL G3A (N=35) FL G3B (N=10)	Anthracycline-based regimen (53%) Non-Anthracycline-based (31%) Not available (16%)	24mo	No difference in OS between FL G3A and G3B No survival improvement or potential curability after anthracyclines-based chemotherapy
Ganti et al., 2006[139]	FL G1 (N=59) FL G2 (N=135) FL G3 (N=136)	Anthracycline-based chemotherapy	9 years	FL G3A and 3B had a similar outcome, but those with a diffuse component of >50% had the worst outcome. OS and EFS curves showed a plateau for patients younger than 60 years of age at diagnosis
Shustik et al., 2011[23]	FL G3A (N=139) FL G3B (N=22)	Various initial therapies. Anthracycline-based regimen in 82% of G3B versus 36% of G3A One-third of the entire cohort received rituximab	45 mo	No difference in outcome between G3A and G3B and no plateau of the OS curves Analysis limited to FL G3A patients showed no evidence of curability with anthracycline-based therapy
Wahlin et al, 2012[24]	FL G1–2 (N=345) FL G3A (N=94) FL G3B (N=23)	Anthracyclines and Rituximab (7%) Anthracycline without rituximab (27%) Rituximab only (7%) Single alkylator (28%) Other regimens (3%) Local radiation (19%) Never treated (9%)	10 years	FL G3B patients reached a plateau OS curve beyond 5 years if treated with anthracyclines, while FL G1–2 and G3A patients continued to relapse beyond 5 years. FL G1–2 and G3A seemed equally indolent, with indistinguishable clinical courses, even in patients receiving anthracyclines.
Koch et al, 2016[140]	FL G3A (N=47) FL G3B (N=14)	Anthracycline and rituximab (52%) Anthracycline without rituximab (48%)	6.9 years	FL G3A and G3B had similar PFS and OS at 5 years, showing a survival curves plateau after 6 years
Mercadal et al., 2016[27]	FL G3A (N=88) G1–2 (N=369)	Anthracycline and rituximab (76%) Anthracycline without rituximab (24%)	5 years	no difference in terms of OS and PFS between the FL G3A and G1–2 cohorts in patients treated with anthracyclines

FL: Follicular lymphoma; G: grade; OS: Overall survival; FLCL: Follicular large cell lymphoma (characterized by more than 15 centroblastic cells per h.p.f. according to International Working Formulation); FFS: Failure Free Survival; RT: Radiotherapy; PFS: Progression Free Survival; mo: months.

## References

[1] Conconi A (2010). Patterns of survival of follicular lymphomas at a single institution through three decades. Leuk Lymphoma.

[2] Tan D (2013). Improvements in observed and relative survival in follicular grade 1–2 lymphoma during 4 decades: the Stanford University experience. Blood.

[3] Casulo C (2014). Disease Characteristics, Treatment Patterns, and Outcomes of Follicular Lymphoma in Patients 40 Years of Age and Younger: An Analysis from the National LymphoCare Study.

[4] Junlen HR (2015). Follicular lymphoma in Sweden: nationwide improved survival in the rituximab era, particularly in elderly women: a Swedish Lymphoma Registry study. Leukemia.

[5] Armitage JO, Longo DL (2016). Is watch and wait still acceptable for patients with low-grade follicular lymphoma?. Blood.

[6] Conconi A (2015). Life expectancy of young adults with follicular lymphoma. Ann Oncol.

[7] Casulo C (2015). Disease characteristics, treatment patterns, and outcomes of follicular lymphoma in patients 40 years of age and younger: an analysis from the National Lymphocare Studydagger. Ann Oncol.

[8] Cheah CY (2016). Factors influencing outcome in advanced stage, low-grade follicular lymphoma treated at MD Anderson Cancer Center in the rituximab era. Ann Oncol.

[9] Friedberg JW (2006). Potential long-term toxicities should influence the choice of therapy for indolent non-Hodgkin’s lymphoma. Haematologica.

[10] Nooka AK (2013). Examination of the follicular lymphoma international prognostic index (FLIPI) in the National LymphoCare study (NLCS): a prospective US patient cohort treated predominantly in community practices. Ann Oncol.

[11] Pastore A (2015). Integration of gene mutations in risk prognostication for patients receiving first-line immunochemotherapy for follicular lymphoma: a retrospective analysis of a prospective clinical trial and validation in a population-based registry. Lancet Oncol.

[12] Plancarte F (2006). Follicular lymphoma in early stages: high risk of relapse and usefulness of the Follicular Lymphoma International Prognostic Index to predict the outcome of patients. Eur J Haematol.

[13] Federico M (2009). Follicular lymphoma international prognostic index 2: a new prognostic index for follicular lymphoma developed by the international follicular lymphoma prognostic factor project. J Clin Oncol.

[14] Decaudin D (1999). Low-grade stage III-IV follicular lymphoma: multivariate analysis of prognostic factors in 484 patients--a study of the groupe d’Etude des lymphomes de l’Adulte. J Clin Oncol.

[15] McClanahan F (2010). Clinical outcome of patients with follicular lymphoma and bulky disease after rituximab-CHOP immunochemotherapy with and without consolidating radiotherapy. Eur J Haematol.

[16] Cheson BD (2014). Recommendations for initial evaluation, staging, and response assessment of Hodgkin and non-Hodgkin lymphoma: the Lugano classification. J Clin Oncol.

[17] Dreyling M (2014). Newly diagnosed and relapsed follicular lymphoma: ESMO Clinical Practice Guidelines for diagnosis, treatment and follow-up. Ann Oncol.

[18] Swerdlow SH (2016). The 2016 revision of the World Health Organization classification of lymphoid neoplasms. Blood.

[19] Gangatharan SA (2015). Clinical characteristics and early treatment outcomes of follicular lymphoma in young adults. Br J Haematol.

[20] Jager U (2012). Rituximab serum concentrations during immuno-chemotherapy of follicular lymphoma correlate with patient gender, bone marrow infiltration and clinical response. Haematologica.

[21] Salles G (2011). Rituximab maintenance for 2 years in patients with high tumour burden follicular lymphoma responding to rituximab plus chemotherapy (PRIMA): a phase 3, randomised controlled trial. Lancet.

[22] Chau I (2003). Outcome of follicular lymphoma grade 3: is anthracycline necessary as front-line therapy?. Br J Cancer.

[23] Shustik J (2011). Follicular non-Hodgkin lymphoma grades 3A and 3B have a similar outcome and appear incurable with anthracycline-based therapy. Ann Oncol.

[24] Wahlin BE (2012). Clinical significance of the WHO grades of follicular lymphoma in a population-based cohort of 505 patients with long follow-up times. Br J Haematol.

[25] Rodriguez J (2000). Follicular large cell lymphoma: long-term follow-up of 62 patients treated between 1973–1981. Ann Oncol.

[26] Miller TP (1997). Follicular lymphomas: do histologic subtypes predict outcome?. Hematol Oncol Clin North Am.

[27] Mercadal S (2016). Clinico-biological features, treatment and survival of 457 patients with histological Grades 3A and 1–2 follicular lymphoma mostly treated with immunochemotherapy. Br J Haematol.

[28] Roulland S (2014). t(14;18) Translocation: A predictive blood biomarker for follicular lymphoma. J Clin Oncol.

[29] Leich E (2016). Similar clinical features in follicular lymphomas with and without breaks in the BCL2 locus. Leukemia.

[30] Katzenberger T (2009). A distinctive subtype of t(14;18)-negative nodal follicular non-Hodgkin lymphoma characterized by a predominantly diffuse growth pattern and deletions in the chromosomal region 1p36. Blood.

[31] Glas AM (2005). Gene expression profiling in follicular lymphoma to assess clinical aggressiveness and to guide the choice of treatment. Blood.

[32] Mir MA (2015). Elevated serum levels of IL-2R, IL-1RA, and CXCL9 are associated with a poor prognosis in follicular lymphoma. Blood.

[33] Wilcox RA (2015). Cutaneous B-cell lymphomas: 2015 update on diagnosis, risk-stratification, and management. Am J Hematol.

[34] Kiess AP, Yahalom J (2013). Primary follicular lymphoma of the gastrointestinal tract: effect of stage, symptoms and treatment choice on outcome. Leuk Lymphoma.

[35] Harada A (2016). Radiation therapy for localized duodenal low-grade follicular lymphoma. J Radiat Res.

[36] Montoto S, Fitzgibbon J (2011). Transformation of indolent B-cell lymphomas. J Clin Oncol.

[37] Montoto S (2007). Risk and clinical implications of transformation of follicular lymphoma to diffuse large B-cell lymphoma. J Clin Oncol.

[38] Conconi A (2012). Incidence, risk factors and outcome of histological transformation in follicular lymphoma. Br J Haematol.

[39] Link BK (2013). Rates and outcomes of follicular lymphoma transformation in the immunochemotherapy era: a report from the University of Iowa/MayoClinic Specialized Program of Research Excellence Molecular Epidemiology Resource. J Clin Oncol.

[40] Brice P (1997). Comparison in low-tumor-burden follicular lymphomas between an initial no-treatment policy, prednimustine, or interferon alfa: a randomized study from the Groupe d’Etude des Lymphomes Folliculaires. Groupe d’Etude des Lymphomes de l’Adulte. J Clin Oncol.

[41] Young RC (1988). The treatment of indolent lymphomas: watchful waiting v aggressive combined modality treatment. Semin Hematol.

[42] Al-tourah A, GK, Hoskins PJ (2006). The impact of initial treatment of advanced stage indolent lymphoma on the risk of transformation. J Clin Oncol.

[43] Gine E (2006). The Follicular Lymphoma International Prognostic Index (FLIPI) and the histological subtype are the most important factors to predict histological transformation in follicular lymphoma. Ann Oncol.

[44] Salles G, Fea SJ (2013). Updated 6 years follow-up of the PRIMA Study confirms the benefit of 2-years Rituximab maintenance in follicular lymphoma patients responding to frontline immunochemotherapy. Haematologica.

[45] Ardeshna KM (2014). Rituximab versus a watch-and-wait approach in patients with advanced-stage, asymptomatic, non-bulky follicular lymphoma: an open-label randomised phase 3 trial. Lancet Oncol.

[46] Wagner-Johnston ND (2015). Outcomes of transformed follicular lymphoma in the modern era: a report from the National LymphoCare Study (NLCS). Blood.

[47] Jerusalem G (2001). Positron emission tomography (PET) with 18F-fluorodeoxyglucose (18F-FDG) for the staging of low-grade non-Hodgkin’s lymphoma (NHL). Ann Oncol.

[48] Karam M (2006). Role of fluorine-18 fluoro-deoxyglucose positron emission tomography scan in the evaluation and follow-up of patients with low-grade lymphomas. Cancer.

[49] Wohrer S (2006). 18F-fluoro-deoxy-glucose positron emission tomography (18F-FDG-PET) visualizes follicular lymphoma irrespective of grading. Ann Oncol.

[50] Tychyj-Pinel C (2014). PET/CT assessment in follicular lymphoma using standardized criteria: central review in the PRIMA study. Eur J Nucl Med Mol Imaging.

[51] Noy A (2009). The majority of transformed lymphomas have high standardized uptake values (SUVs) on positron emission tomography (PET) scanning similar to diffuse large B-cell lymphoma (DLBCL). Ann Oncol.

[52] Novelli S (2015). PET/CT Assessment of Follicular Lymphoma and High Grade B Cell Lymphoma - Good Correlation with Clinical and Histological Features at Diagnosis. Adv Clin Exp Med.

[53] Wondergem MJ (2015). 18F-FDG or 3′-deoxy-3′-18F-fluorothymidine to detect transformation of follicular lymphoma. J Nucl Med.

[54] Bodet-Milin C (2008). Investigation of FDG-PET/CT imaging to guide biopsies in the detection of histological transformation of indolent lymphoma. Haematologica.

[55] Karam M (2011). Features of large cell transformation of indolent lymphomas as observed on sequential PET/CT. Nucl Med Commun.

[56] Trotman J (2011). Positron emission tomography-computed tomography (PET-CT) after induction therapy is highly predictive of patient outcome in follicular lymphoma: analysis of PET-CT in a subset of PRIMA trial participants. J Clin Oncol.

[57] Luminari S (2014). The prognostic role of post-induction FDG-PET in patients with follicular lymphoma: a subset analysis from the FOLL05 trial of the Fondazione Italiana Linfomi (FIL). Ann Oncol.

[58] Dupuis J (2012). Impact of [(18)F]fluorodeoxyglucose positron emission tomography response evaluation in patients with high-tumor burden follicular lymphoma treated with immunochemotherapy: a prospective study from the Groupe d’Etudes des Lymphomes de l’Adulte and GOELAMS. J Clin Oncol.

[59] Trotman J (2014). Prognostic value of PET-CT after first-line therapy in patients with follicular lymphoma: a pooled analysis of central scan review in three multicentre studies. Lancet Haematol.

[60] Barrington SF (2014). Role of imaging in the staging and response assessment of lymphoma: consensus of the International Conference on Malignant Lymphomas Imaging Working Group. J Clin Oncol.

[61] Meignan M (2016). Baseline Metabolic Tumor Volume Predicts Outcome in High-Tumor-Burden Follicular Lymphoma: A Pooled Analysis of Three Multicenter Studies. J Clin Oncol.

[62] Luminari S (2016). Positron emission tomography response and minimal residual disease impact on progression-free survival in patients with follicular lymphoma. A subset analysis from the FOLL05 trial of the Fondazione Italiana Linfomi. Haematologica.

[63] Ladetto M (2002). High rate of clinical and molecular remissions in follicular lymphoma patients receiving high-dose sequential chemotherapy and autografting at diagnosis: a multicenter, prospective study by the Gruppo Italiano Trapianto Midollo Osseo (GITMO). Blood.

[64] Galimberti S (2003). Quantitative molecular evaluation in autotransplant programs for follicular lymphoma: efficacy of in vivo purging by Rituximab. Bone Marrow Transplant.

[65] Apostolidis J (2000). High-dose therapy with autologous bone marrow support as consolidation of remission in follicular lymphoma: long-term clinical and molecular follow-up. J Clin Oncol.

[66] Corradini P (2004). Long-term follow-up of indolent lymphoma patients treated with high-dose sequential chemotherapy and autografting: evidence that durable molecular and clinical remission frequently can be attained only in follicular subtypes. J Clin Oncol.

[67] Lopez-Guillermo A (1998). The clinical significance of molecular response in indolent follicular lymphomas. Blood.

[68] Rambaldi A (2002). Monitoring of minimal residual disease after CHOP and rituximab in previously untreated patients with follicular lymphoma. Blood.

[69] Hirt C (2008). Rapid and sustained clearance of circulating lymphoma cells after chemotherapy plus rituximab: clinical significance of quantitative t(14;18) PCR monitoring in advanced stage follicular lymphoma patients. Br J Haematol.

[70] Schmitt C (2006). One single dose of rituximab added to a standard regimen of CHOP in primary treatment of follicular lymphoma appears to result in a high clearance rate from circulating bcl-2/IgH positive cells: Is the end of molecular monitoring near?. Leuk Res.

[71] Paszkiewicz-Kozik E (2009). Presence of t(14;18) positive cells in blood and bone marrow does not predict outcome in follicular lymphoma. Med Oncol.

[72] van Oers MH (2010). BCL-2/IgH polymerase chain reaction status at the end of induction treatment is not predictive for progression-free survival in relapsed/resistant follicular lymphoma: results of a prospective randomized EORTC 20981 phase III intergroup study. J Clin Oncol.

[73] Ladetto M (2013). Persistence of minimal residual disease in bone marrow predicts outcome in follicular lymphomas treated with a rituximab-intensive program. Blood.

[74] Galimberti S (2014). Minimal residual disease after conventional treatment significantly impacts on progression-free survival of patients with follicular lymphoma: the FIL FOLL05 trial. Clin Cancer Res.

[75] Guadagnolo BA (2006). Long-term outcome and mortality trends in early-stage, Grade 1–2 follicular lymphoma treated with radiation therapy. Int J Radiat Oncol Biol Phys.

[76] Petersen PM, GR, Tsang R (2004). Long term outcome in stage I and II follicular lymphoma following treatent with involved field radiation therapy alone. J Clin Oncol.

[77] Pugh TJ (2010). Improved survival in patients with early stage low-grade follicular lymphoma treated with radiation: a Surveillance, Epidemiology, and End Results database analysis. Cancer.

[78] Advani R, Rosenberg SA, Horning SJ (2004). Stage I and II follicular non-Hodgkin’s lymphoma: long-term follow-up of no initial therapy. J Clin Oncol.

[79] Friedberg JW (2009). Follicular lymphoma in the United States: first report of the national LymphoCare study. J Clin Oncol.

[80] Barzenje DA (2015). Radiotherapy Compared to Other Strategies in the Treatment of Stage I/II Follicular Lymphoma: A Study of 404 Patients with a Median Follow-Up of 15 Years. PLoS One.

[81] Michallet AS (2013). Early stage follicular lymphoma: what is the clinical impact of the first-line treatment strategy?. J Hematol Oncol.

[82] Sancho JM (2015). The long term follow-up of early stage follicular lymphoma treated with radiotherapy, chemotherapy or combined modality treatment. Leuk Res.

[83] Bains P (2013). Incidence of transformation to aggressive lymphoma in limited-stage follicular lymphoma treated with radiotherapy. Ann Oncol.

[84] Kelsey SM (1994). A British National Lymphoma Investigation randomised trial of single agent chlorambucil plus radiotherapy versus radiotherapy alone in low grade, localised non-Hodgkins lymphoma. Med Oncol.

[85] Yahalom J (1993). Adjuvant cyclophosphamide, doxorubicin, vincristine, and prednisone chemotherapy after radiation therapy in stage I low-grade and intermediate-grade non-Hodgkin lymphoma. Results of a prospective randomized study. Cancer.

[86] Seymour JF (2003). Long-term follow-up of a prospective study of combined modality therapy for stage I-II indolent non-Hodgkin’s lymphoma. J Clin Oncol.

[87] Skvortsova I (2005). Pretreatment with rituximab enhances radiosensitivity of non-Hodgkin’s lymphoma cells. J Radiat Res.

[88] Ruella M (2016). Addition of Rituximab to Involved-Field Radiation Therapy Prolongs Progression-free Survival in Stage I-II Follicular Lymphoma: Results of a Multicenter Study. Int J Radiat Oncol Biol Phys.

[89] Janikova A (2015). Radiotherapy with rituximab may be better than radiotherapy alone in first-line treatment of early-stage follicular lymphoma: is it time to change the standard strategy?. Leuk Lymphoma.

[90] Mondello P (2014). Radiotherapy for stage I/II follicular lymphoma (FL): is it time for a re-appraisal?. Anticancer Res.

[91] Ardeshna KM (2003). Long-term effect of a watch and wait policy versus immediate systemic treatment for asymptomatic advanced-stage non-Hodgkin lymphoma: a randomised controlled trial. Lancet.

[92] Rummel MJ (2013). Bendamustine plus rituximab versus CHOP plus rituximab as first-line treatment for patients with indolent and mantle-cell lymphomas: an open-label, multicentre, randomised, phase 3 non-inferiority trial. Lancet.

[93] Portlock CS, Rosenberg SA (1979). No initial therapy for stage III and IV non-Hodgkin’s lymphomas of favorable histologic types. Ann Intern Med.

[94] Horning SJ, Rosenberg SA (1984). The natural history of initially untreated low-grade non-Hodgkin’s lymphomas. N Engl J Med.

[95] Witzig TE (2005). Rituximab therapy for patients with newly diagnosed, advanced-stage, follicular grade I non-Hodgkin’s lymphoma: a phase II trial in the North Central Cancer Treatment Group. J Clin Oncol.

[96] Colombat P (2001). Rituximab (anti-CD20 monoclonal antibody) as single first-line therapy for patients with follicular lymphoma with a low tumor burden: clinical and molecular evaluation. Blood.

[97] Martinelli G (2010). Long-term follow-up of patients with follicular lymphoma receiving single-agent rituximab at two different schedules in trial SAKK 35/98. J Clin Oncol.

[98] Taverna C (2016). Rituximab Maintenance for a Maximum of 5 Years After Single-Agent Rituximab Induction in Follicular Lymphoma: Results of the Randomized Controlled Phase III Trial SAKK 35/03. J Clin Oncol.

[99] Hiddemann W (2005). Frontline therapy with rituximab added to the combination of cyclophosphamide, doxorubicin, vincristine, and prednisone (CHOP) significantly improves the outcome for patients with advanced-stage follicular lymphoma compared with therapy with CHOP alone: results of a prospective randomized study of the German Low-Grade Lymphoma Study Group. Blood.

[100] Marcus R (2008). Phase III study of R-CVP compared with cyclophosphamide, vincristine, and prednisone alone in patients with previously untreated advanced follicular lymphoma. J Clin Oncol.

[101] Herold M (2007). Rituximab added to first-line mitoxantrone, chlorambucil, and prednisolone chemotherapy followed by interferon maintenance prolongs survival in patients with advanced follicular lymphoma: an East German Study Group Hematology and Oncology Study. J Clin Oncol.

[102] Schulz H (2007). Immunochemotherapy with rituximab and overall survival in patients with indolent or mantle cell lymphoma: a systematic review and meta-analysis. J Natl Cancer Inst.

[103] Federico M (2013). R-CVP versus R-CHOP versus R-FM for the initial treatment of patients with advanced-stage follicular lymphoma: results of the FOLL05 trial conducted by the Fondazione Italiana Linfomi. J Clin Oncol.

[104] Flinn IW (2014). Randomized trial of bendamustine-rituximab or R-CHOP/R-CVP in first-line treatment of indolent NHL or MCL: the BRIGHT study. Blood.

[105] Ghielmini M (2004). Prolonged treatment with rituximab in patients with follicular lymphoma significantly increases event-free survival and response duration compared with the standard weekly × 4 schedule. Blood.

[106] Kahl BS (2014). Rituximab extended schedule or re-treatment trial for low-tumor burden follicular lymphoma: eastern cooperative oncology group protocol e4402. J Clin Oncol.

[107] Leonard JP (2015). Randomized Trial of Lenalidomide Alone Versus Lenalidomide Plus Rituximab in Patients With Recurrent Follicular Lymphoma: CALGB 50401 (Alliance). J Clin Oncol.

[108] Fowler NH (2014). Safety and activity of lenalidomide and rituximab in untreated indolent lymphoma: an open-label, phase 2 trial. Lancet Oncol.

[109] Peter Martin S-HJ, Johnson Jeffrey L, Pitcher Brandy, Elstrom Rebecca L, Bartlett Nancy, Blum Kristie A, Richards Kristy L, Leonard John, Bruce D (2014). Cheson CALGB 50803 (Alliance), A phase II trial of lenalidomide plus rituximab in patients with previously untreated follicular lymphoma. Journal of Clinical Oncology.

[110] Kimby E, MG (2014). Rituximab Plus Lenalidomide Improves the Complete Remission Rate in Comparison with Rituximab Monotherapy in Untreated Follicular Lymphoma Patients in Need of Therapy. Primary Endpoint Analysis of the Randomized Phase-2 Trial SAKK 35/10. Blood (ASH Annual Meeting Abstracts).

[111] Zucca E (2015). Independent review of CT responses in the trial SAKK 35/10 comparing rituximab with or without lenalidomide in untreated FL patients in need of therapy. Hematol Oncol.

[112] Kimby E, Zucca E (2016). Rituximab Plus Lenalidomide Versus Rituximab Monotherapy in Untreated Follicular Lymphoma Patients in Need of Therapy. First Analysis of Survival Endpoints of the Randomized Phase-2 Trial SAKK 35/10. Blood (ASH Annual Meeting Abstracts).

[113] Marcus RE (2016). Obinutuzumab-Based Induction and Maintenance Prolongs Progression-Free Survival (PFS) in Patients with Previously Untreated Follicular Lymphoma: Primary Results of the Randomized Phase 3 GALLIUM StudyClinically Relevant Abstract. Blood (ASH Annual Meeting Abstracts).

[114] Hainsworth JD (2002). Rituximab as first-line and maintenance therapy for patients with indolent non-hodgkin’s lymphoma. J Clin Oncol.

[115] van Oers MH (2006). Rituximab maintenance improves clinical outcome of relapsed/resistant follicular non-Hodgkin lymphoma in patients both with and without rituximab during induction: results of a prospective randomized phase 3 intergroup trial. Blood.

[116] Vidal L (2009). Rituximab maintenance for the treatment of patients with follicular lymphoma: systematic review and meta-analysis of randomized trials. J Natl Cancer Inst.

[117] Hochster H (2009). Maintenance rituximab after cyclophosphamide, vincristine, and prednisone prolongs progression-free survival in advanced indolent lymphoma: results of the randomized phase III ECOG1496 Study. J Clin Oncol.

[118] Maloney DG (1999). Preclinical and phase I and II trials of rituximab. Semin Oncol.

[119] Berinstein NL (1998). Association of serum Rituximab (IDEC-C2B8) concentration and anti-tumor response in the treatment of recurrent low-grade or follicular non-Hodgkin’s lymphoma. Ann Oncol.

[120] Lenz G (2004). Myeloablative radiochemotherapy followed by autologous stem cell transplantation in first remission prolongs progression-free survival in follicular lymphoma: results of a prospective, randomized trial of the German Low-Grade Lymphoma Study Group. Blood.

[121] Sebban C (2006). Standard chemotherapy with interferon compared with CHOP followed by high-dose therapy with autologous stem cell transplantation in untreated patients with advanced follicular lymphoma: the GELF-94 randomized study from the Groupe d’Etude des Lymphomes de l’Adulte (GELA). Blood.

[122] Gyan E (2009). High-dose therapy followed by autologous purged stem cell transplantation and doxorubicin-based chemotherapy in patients with advanced follicular lymphoma: a randomized multicenter study by the GOELAMS with final results after a median follow-up of 9 years. Blood.

[123] Ladetto M (2008). Prospective, multicenter randomized GITMO/IIL trial comparing intensive (R-HDS) versus conventional (CHOP-R) chemoimmunotherapy in high-risk follicular lymphoma at diagnosis: the superior disease control of R-HDS does not translate into an overall survival advantage. Blood.

[124] Schaaf M (2012). High-dose therapy with autologous stem cell transplantation versus chemotherapy or immuno-chemotherapy for follicular lymphoma in adults. Cochrane Database Syst Rev.

[125] Kaminski MS (2005). 131I-tositumomab therapy as initial treatment for follicular lymphoma. N Engl J Med.

[126] Ibatici A (2014). Safety and efficacy of (90) yttrium-ibritumomab-tiuxetan for untreated follicular lymphoma patients. An Italian cooperative study. Br J Haematol.

[127] Illidge TM (2014). Fractionated (9)(0)Y-ibritumomab tiuxetan radioimmunotherapy as an initial therapy of follicular lymphoma: an international phase II study in patients requiring treatment according to GELF/BNLI criteria. J Clin Oncol.

[128] Scholz CW (2013). (90)Yttrium-ibritumomab-tiuxetan as first-line treatment for follicular lymphoma: 30 months of follow-up data from an international multicenter phase II clinical trial. J Clin Oncol.

[129] Press OW (2003). A phase 2 trial of CHOP chemotherapy followed by tositumomab/iodine I 131 tositumomab for previously untreated follicular non-Hodgkin lymphoma: Southwest Oncology Group Protocol S9911. Blood.

[130] Leonard JP (2005). Abbreviated chemotherapy with fludarabine followed by tositumomab and iodine I 131 tositumomab for untreated follicular lymphoma. J Clin Oncol.

[131] Hainsworth JD (2009). Rituximab plus short-duration chemotherapy followed by Yttrium-90 Ibritumomab tiuxetan as first-line treatment for patients with follicular non-Hodgkin lymphoma: a phase II trial of the Sarah Cannon Oncology Research Consortium. Clin Lymphoma Myeloma.

[132] Provencio M (2014). Consolidation treatment with Yttrium-90 ibritumomab tiuxetan after new induction regimen in patients with intermediate- and high-risk follicular lymphoma according to the follicular lymphoma international prognostic index: a multicenter, prospective phase II trial of the Spanish Lymphoma Oncology Group. Leuk Lymphoma.

[133] Zinzani PL (2012). A phase II trial of short course fludarabine, mitoxantrone, rituximab followed by (9)(0)Y-ibritumomab tiuxetan in untreated intermediate/high-risk follicular lymphoma. Ann Oncol.

[134] Morschhauser F (2013). 90Yttrium-ibritumomab tiuxetan consolidation of first remission in advanced-stage follicular non-Hodgkin lymphoma: updated results after a median follow-up of 7.3 years from the International, Randomized, Phase III First-LineIndolent trial. J Clin Oncol.

[135] Press OW (2013). Phase III randomized intergroup trial of CHOP plus rituximab compared with CHOP chemotherapy plus (131)iodine-tositumomab for previously untreated follicular non-Hodgkin lymphoma: SWOG S0016. J Clin Oncol.

[136] Lopez-Guillermo A, Canales M, Dlouhy I, Briones J, Caballero D, Sancho J (2013). A randomized Phase II study comparing consolidation with a single dose of 90Y ibritumomab tiuxetan (Zevalin®) (Z) vs. maintenance with rituximab (R) for two years in patients with newly diagnosed follicular lymphoma (FL) responding to R-CHOP. Preliminary results at 36 months from randomization. Blood (ASH Annual Meeting Abstracts).

[137] Chen Q (2015). Comparing the cost-effectiveness of rituximab maintenance and radioimmunotherapy consolidation versus observation following first-line therapy in patients with follicular lymphoma. Value Health.

[138] Hsi ED (2004). A clinicopathologic evaluation of follicular lymphoma grade 3A versus grade 3B reveals no survival differences. Arch Pathol Lab Med.

[139] Ganti AK (2006). Patients with grade 3 follicular lymphoma have prolonged relapse-free survival following anthracycline-based chemotherapy: the Nebraska Lymphoma Study Group Experience. Ann Oncol.

[140] Koch K (2016). Clinical, pathological and genetic features of follicular lymphoma grade 3A: a joint analysis of the German low-grade and high-grade lymphoma study groups GLSG and DSHNHL. Ann Oncol.

